# Nutrition in Pediatric Gastroenterology

**DOI:** 10.3390/nu13061965

**Published:** 2021-06-08

**Authors:** Claudio Romano, Valeria Dipasquale

**Affiliations:** Pediatric Gastroenterology and Cystic Fibrosis Unit, Department of Human Pathology in Adulthood and Childhood “G. Barresi”, University of Messina, 98125 Messina, Italy; dipasquale.valeria@libero.it

Nutrition and intestinal function are strictly interrelated. The main role of the gut is to digest and absorb nutrients in order to maintain life and well-being. Consequently, chronic gastrointestinal disease commonly results in malnutrition and increased morbidity and mortality. Furthermore, chronic malnutrition impairs digestive and absorptive function because food and nutrients are not only the major trophic factors in the gut but also contribute to the structure and functioning of digestive enzymes and absorptive cells. Finally, specific nutrients can induce or prevent gastrointestinal disease (for example, gluten-sensitive enteropathy, or the role of dietary antigens and elemental diets in inducing remission in patients with Crohn’s disease). In conclusion, nutrition has both a supportive and therapeutic role in the management of many gastrointestinal diseases. All these aspects are highly relevant during infancy and childhood, dynamic phases of life characterized by rapid growth, development and developmental plasticity, in which adequate amounts and compositions of substrates both in health and disease are fundamental for growth, functional outcomes and long-term health.

This Special Issue of *Nutrients*, “Nutrition and Pediatric Gastroenterology”, contains three original publications and five reviews investigating the specific aspects of nutrition that could play a role in gastrointestinal disease in infancy and childhood, both functional and nonfunctional.

The first review published in this Special Issue provides a comprehensive overview of the possible role of diet in the pathogenesis and management of the most common gastrointestinal chronic diseases, both organic (i.e., inflammatory bowel diseases) and functional (i.e., chronic constipation, irritable bowel syndrome) [1]. Moreover, the relations between diet, microbiota and inflammation are highlighted.

Nutritional status and its modifications are pointed out by two review papers [2,3]. The review published by our group summarizes the new definitions and currently used classification of acute malnutrition, as well as the diagnostic assessment and treatment, which can be conducted in primary care with nutrition-specific interventions in most cases [2]. Additionally, Diamanti et al. [3] present a new insight for the definition and management of failure to thrive (FTT), integrating the “classical” anthropometric criteria for definition and treatment with a more complete definition which takes into account both the clinical and anthropometric worsening, and the overall nutrition status, including the micronutrient status. Notably, some predisposing conditions for FTT and micronutrient deficiencies are explored, such as elimination diets (in food allergies, lactose intolerance, vegetarian/vegan diet), family dysfunction with food insecurity and avoidant/restrictive food intake disorders. 

Two papers focus on celiac disease (CD) [4,5]. The international retrospective study conducted by Sansotta et al. [4] investigated the effects of a gluten-free diet on body mass index (BMI) and growth parameters in pediatric patients with CD. The different impacts of a gluten-free diet are described in two groups of CD children followed in two different countries, i.e., Italy and the USA. A significant change in BMI z-scores in pediatric CD groups following the gluten-free diet was not found, though both CD populations had an increase in height standard deviation (SD) and weight SD. The proportion of overweight CD children on the gluten-free diet increased from 6% to 9% in Italy, whereas in the US CD group, the percentage of overweight CD children decreased from 17% to 12% on the gluten-free diet. The rising BMI, whether desired or undesired after treatment of childhood CD, is probably multifactorial and related to lifestyle and cultural differences. Concerning CD, the review by Stefanelli et al. [5] describes the problem of iron deficiency anemia (IDA) as a common CD sign, discussing the main pathogenic mechanisms and the possible causes of its persistence after adopting a correct gluten-free diet, as well as the therapeutic implications.

Contributions related to both immunoglobulin E (IgE)-mediated and non-IgE-mediated gastrointestinal food allergic disorders have been provided [6,7]. One review explores the distinctive clinical features of non-IgE-mediated food allergies—such as food protein-induced enterocolitis syndrome (FPIES), food protein-induced enteropathy (FPE) and food protein-induced allergic proctocolitis (FPIAP)—which may present with gastrointestinal symptoms in children, discussing pathogenesis, management and currents gaps in the diagnosis and medical assessment [6]. The study by Vandenplas et al. [7] focused on cow’s milk allergy and analyzed the inter-rater variability between a pediatrician and parents, and day to day variability of the Cow’s Milk-related Symptom Score (CoMiSS^TM^). The CoMiSS^TM^ is a symptom-based score aimed at evaluating evolution during a therapeutic intervention (awareness tool). The study found an excellent agreement between CoMiSS^TM^ in presumed healthy infants provided by a pediatrician or parents, without the need to provide any special training to the parents.

The Special Issue also includes the results of a double-blind, randomized placebo-controlled clinical trial, which evaluated the efficacy of a probiotic (*Lactobacillus reuteri* DSM 17938), a prebiotic (agave inulin) and a synbiotic on the stool characteristics in 37 children with cerebral palsy and chronic constipation [8]. Indeed, the most common gastrointestinal condition in children with cerebral palsy is constipation, which seems to be a multifactorial pathogenesis, including the dysbiosis of the gut. Increasing evidence supports the use of synbiotics, prebiotics and probiotics in the treatment of functional constipation. Probiotics are live microorganisms that, when administered in adequate amounts, confer a health benefit to the host. Prebiotics are non-digestible food ingredients that selectively stimulate the growth and/or activity of some bacteria in the colon. A synbiotic is a mixture of probiotics and prebiotics. Targeting treatments for dysbiosis with probiotics, prebiotics and synbiotics improve clinical symptoms, promote the recovery of intestinal flora and may be a new option that is significantly useful for the treatment of chronic constipation. In this clinical trial, the probiotic group showed a significant decrease in stool pH (*p* = 0.014). Stool consistency improved in the prebiotic group (*p* = 0.008). The probiotic, prebiotic and synbiotic groups showed a significant improvement in the history of excessive stool retention, the presence of fecal mass in the rectum and the history of painful defecation. It was therefore concluded that children with cerebral palsy and chronic constipation may benefit from the use of *Lactobacillus reuteri* DSM 17938 and/or agave inulin in terms of stool characteristics compared to placebo.

The present Special Issue provides a summary of the progress in many pediatric gastrointestinal diseases and the importance of nutritional assessment. This is of interest from a clinical and public health perspective. Nevertheless, more studies with larger samples and comparable methods are warranted to understand the actual role of macro- and micronutrients in disease development and health maintenance.

## Data Availability

Not applicable.
